# Age- and sex-dependent differences in the morphology and composition of paraspinal muscles between subjects with and without lumbar degenerative diseases

**DOI:** 10.1186/s12891-022-05692-0

**Published:** 2022-08-01

**Authors:** Rufeng Huang, Fumin Pan, Chao Kong, Shibao Lu

**Affiliations:** 1 Department of Orthopedics, Xuanwu Hospital, Capital Medical University, Beijing, China; 2 National Clinical Research Center for Geriatric Diseases, Beijing, China

**Keywords:** Paraspinal muscles, Low back pain, Lumbar degenerative disease, Asymptomatic subjects, Age- and sex-dependent differences

## Abstract

**Background:**

The quality of the paraspinal muscles has been recommended as a surrogate marker for the evaluation of the severity of the lumbar degenerative diseases (LDD). The purpose of this study is to determine the age- and sex-dependent differences in the morphology and composition of the paraspinal muscles between LDD and asymptomatic subjects.

**Methods:**

We analyzed data from 370 patients and 327 asymptomatic volunteers aged between 18–85 years. The measurement of the cross-sectional area (CSA) of the erector spinae, multifidus, and psoas at the L4/5-disc level was performed by the magnetic resonance imaging (MRI). The fatty infiltration ratio (FI %) of the multifidus and erector spinae was calculated.

**Results:**

FI % of the lumbar paraspinal muscles were significantly and positively correlated with the severity of LDD instead of the CSA. Males had greater CSA than females, and females showed higher FI % than males in the paraspinal muscles. With the increase of age, the CSA of the lumbar paraspinal muscles gradually decreased, and the psoas showed the most significant decreasing trend. However, the FI % gradually increased in both LDD and asymptomatic groups with aging.

**Conclusion:**

Age- and sex-dependent differences were found in the morphology and composition of the paraspinal muscles between subjects with and without LDD. Further long-term follow up investigations and basic studies will continue to confirm the natural history of the paraspinal muscles with aging and their association with LDD.

## Introduction

Low back pain (LBP) is a prevalent complaint among the elderly, which is a significant cause of years lived with disability worldwide [1]. Furthermore, both the medical and the non-medical costs associated with LBP are very high. Intervertebral disc degeneration has long been a leading candidate in the development of spinal diseases [2]. Endean et al. identified disc degeneration, nerve root compression, and hyperintensity zones on the magnetic resonance imaging (MRI) as predictors of LBP [3]. Evidence from other studies suggested that disc herniation and chronic LBP patients showed atrophy of the spinal muscles and infiltration of fatty tissues [4, 5]. Fortin et al. clarified that the morphology and fatty infiltration of the multifidus and psoas is associated with patients’ functional status and symptoms [5]. Jermy et al. found a trend for better outcomes following surgery if pre-operative multifidus quality is better [6].

The lumbar paraspinal muscle plays a critical role in maintaining the coronal and sagittal alignment [7, 8]. Kang et al. [9] observed that the paraspinal muscle volume reduces the pressure exerted on the lumbar spine. By strengthening the paraspinal muscles, the pressure on the lumbar spine can be reduced, and in previous studies, it has been found to have pain-relieving properties [9, 10]. Thus, the atrophy resulting from sarcopenia and fatty infiltration of the paraspinal muscles is a cause of disc degeneration [5]. Recent studies have also demonstrated that fatty infiltration in multifidus and reduced cross-sectional area (CSA) in psoas may contribute to lower functional performance in patients with lumbar spinal stenosis [5, 11]. Additionally, aging is a cause of sarcopenia, which has been recognized with severer functional disability in patients [12, 13]. In patients with lumbar degenerative diseases (LDD), MRI-based evaluation of the paraspinal muscles has been recommended as a surrogate marker [14, 15]. In previous studies, they found that the CSA of the paraspinal muscles are greater among males than females. However, very few imaging studies have examined the differences in the morphology and composition of the paraspinal muscles in LDD subjects with different ages and sexes. Specifically, most attention has been directed at the multifidus instead of at the erector spinae and the psoas [4–7, 11, 15].

The aims of this study are therefore to determine the age- and sex-matched differences in the morphology and composition of paraspinal muscles (the multifidus, the erector spinae, and the psoas) between subjects with and without LDD.

## Materials and methods

### Participants

This retrospective cross-sectional study was approved by the institutional review board of Xuanwu Hospital Capital Medical University. All investigations were carried out in accordance with the relevant guidelines and in accordance with the Declaration of Helsinki. Informed consent was obtained from all subjects prior to the enrollment of the relevant data and publication of identifying information/images in an online open-access publication. Two spinal surgeons diagnosed LDD based on the chief complaint, clinical examinations, and radiological data of patients. The medical records of patients with LDD (including lumbar disc herniation, lumbar spinal stenosis, and lumbar spondylolisthesis) who were between 18 and 85 years old and underwent lumbar decompression and fusion surgery between January 2020 and January 2022, were retrospectively reviewed. Patients with neurological, respiratory, circulatory diseases, lumbar vertebral compression fractures, scoliosis, neuromuscular diseases or previously undergone spinal surgery were excluded [5, 8]. Asymptomatic subjects without LDD between the ages of 18 and 85 years were recruited if they had no current and history of LBP in the past year. The exclusion criteria including lumbar fracture, scoliosis, neuromuscular disease, malignancy, or history of spinal surgery [16]. All participants including LDD and asymptomatic were grouped by sex and age (18–39, or 40–59, or ≥ 60 years old).

### Magnetic resonance imaging

All MRI scans were performed using the 3.0-T scanners (Avanto, Siemens Healthiness, or Signa, GE Healthcare). T2W axial MR images were acquired at the intervertebral disc (IVD) level parallel to the disc from L1–L2 to L5–S1. There were three 4-mm-thick slices of image, including the inferior endplate of the upper vertebra, the middle of IVD, and the superior endplate of the lower vertebra. In this study, the inferior endplate of the upper vertebra at L4-L5 disc level was used to acquire the muscle measurements. DICOM formatted MR images were downloaded for analysis [17].

### Paraspinal muscle parameters

**Fig. 1:**
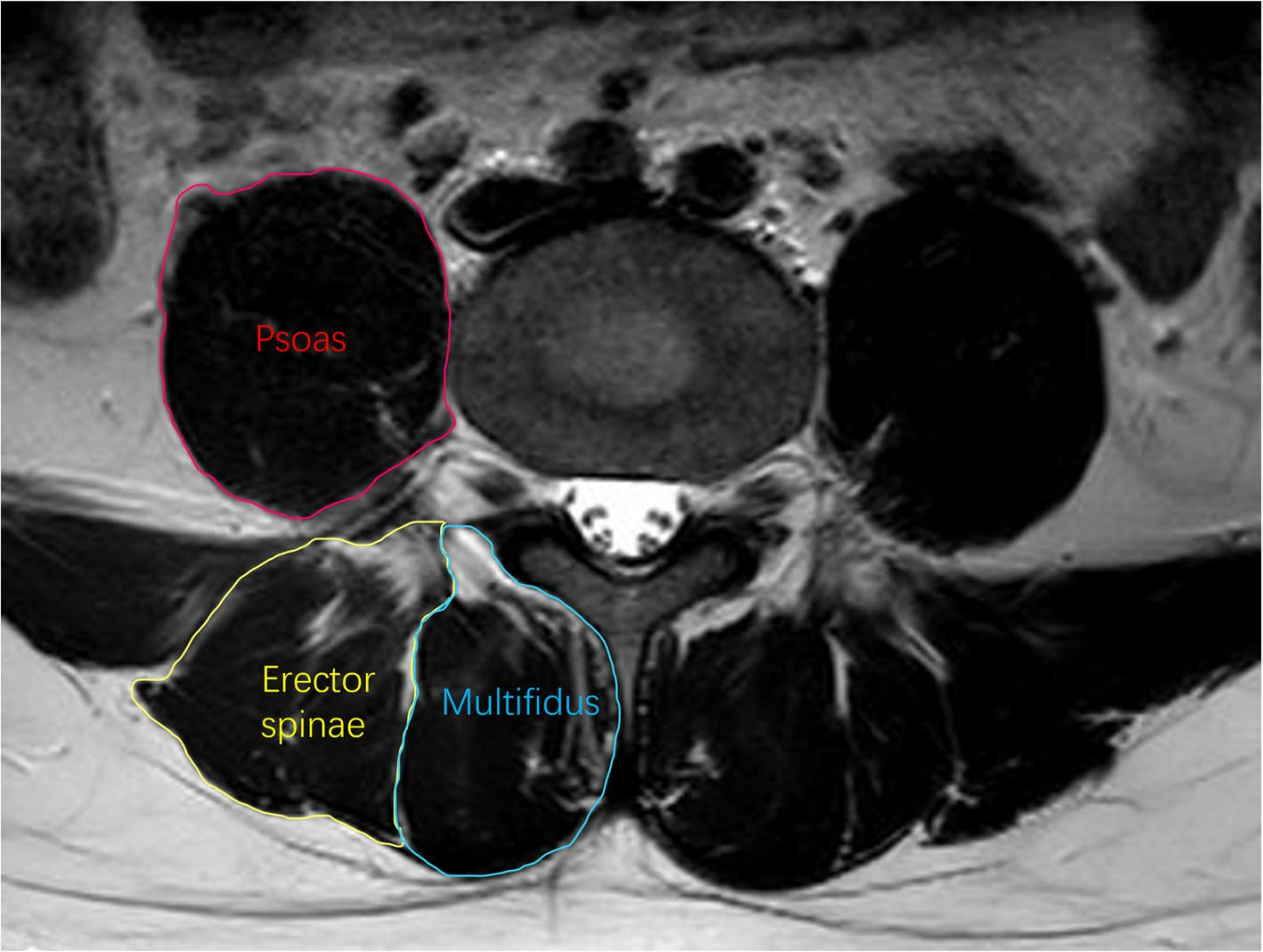
The measurement of multifidus, erector spinae, and psoas

The measurement of the CSA in paraspinal muscles (the multifidus, the erector spinae, and the psoas major) and fatty infiltration ratio (FI %) in the multifidus and the erector spinae were performed by MR images. The image-processing software platform (Image J, National Institutes of Health, Bethesda, MD) was used to trace out the boundary of the multifidus, erector spinae, and psoas (Fig. 1) [15, 18]. FI% was calculated using threshold technique [8]. We randomly selected 100 MR images for two experienced orthopedic surgeons to assess the inter-observer reliability. For evaluating intra-observer reliability, one month after the first measurements of selected images, the second evaluation was conducted again by the same observer. The ICCs were 0.96 for inter-observer reliability and 0.97 for intra-observer reliability.

### Statistical analysis

All statistical analyses were performed using the Statistical Package for the Social Sciences (SPSS, version 26.0, SPSS, Inc, Chicago, IL, USA). Data normality was tested using the Shapiro–Wilk test and variability using the Leneve test. Descriptive statistics of CSA and FI % of the paraspinal muscles were expressed as the mean and standard deviation (SD) or the median (1st quartile, 3rd quartile). Independent-samples T test or Mann–Whitney U test were used to compare the differences of CSA and FI % between sex- and age-matched groups for continuous variables. A P value below 0.05 was considered to indicate a statistically significant difference [5].

## Results

### The baseline characteristics of LDD patients and asymptomatic

Table 1 shows the baseline characteristics of LDD patients and asymptomatic subjects. There was no significant difference in sex (173 [46.76%] VS 160 [48.93%], *p* = 0.567) and BMI (25.70 [23.44, 28.07] VS 25.26 [21.97, 29.10], *p* = 0.200) between two groups. The age of LDD group significantly greater than that of the asymptomatic group (62 [46, 69] VS 44 [34, 61], *p* < 0.001*).

**Table 1:** The baseline characteristics of LDD patients and Asymptomatic

	LDD (n = 370)	Asymptomatic (*n* = 327)	*P* value
Age	62 (46, 69)	44 (34, 61)	< 0.001*
Male (%)	173 (46.76%)	160 (48.93%)	0.567
BMI	25.70 (23.44, 28.07)	25.26 (21.97, 29.10)	0.200

*BMI* Body mass index

^*^ Statistical significance at the level of 0.05

### The differences in the CSA and FI % of the paraspinal muscles among the different age strata and sex in the LDD and asymptomatic groups

**Fig. 2:**
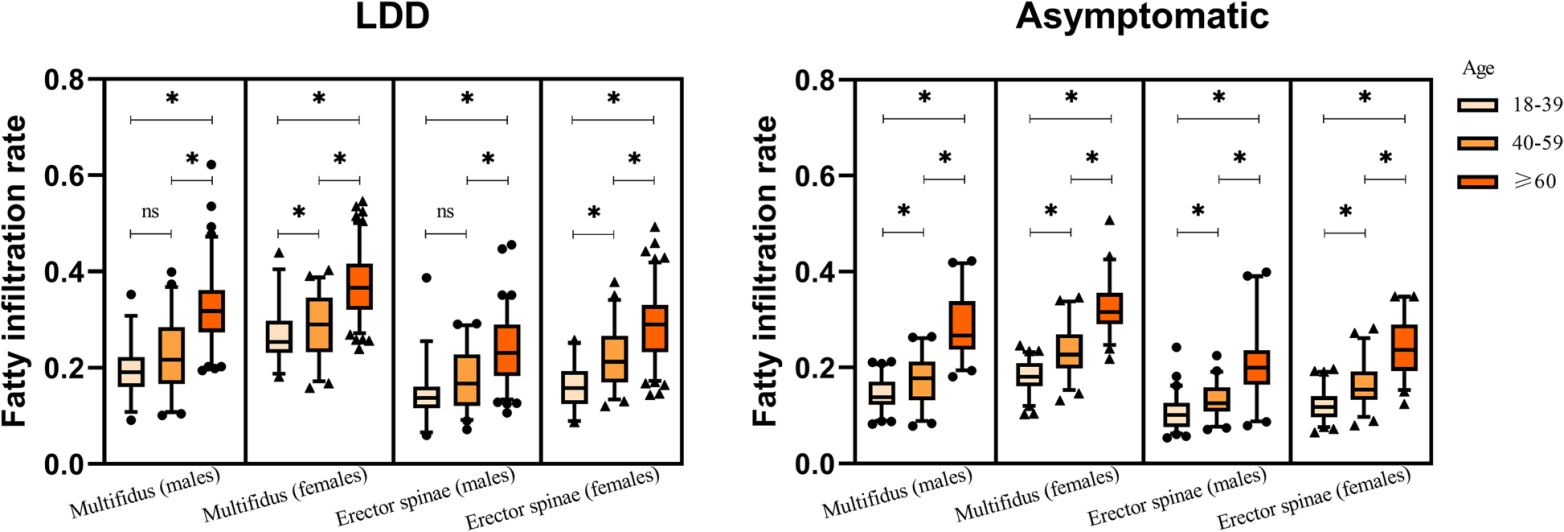
The differences in the fatty infiltration rate of the paraspinal muscles among the different age strata and sex in the LDD (lumbar degenerative disease) and asymptomatic groups. *Statistical significance at the level of 0.05

A total of 370 LDD patients (male 173, female 197) and 327 (male 160, female 167) asymptomatic volunteers were enrolled. Table 2 shows the differences in the CSA and FI % of the paraspinal muscles among the different age strata and sex in the LDD and asymptomatic groups. With the increase of age, the CSA of the lumbar paraspinal muscles gradually decreased, and the FI % gradually increased in both groups. Psoas showed the most significant decreasing trend in CSA with an advancing age in all muscles, followed by erector spinae and multifidus. Compared to the erector spinae, the multifidus showed a greater fat infiltration. In addition, males had greater CSA and lower FI% than females in the paraspinal muscles (Fig. 2).

**Table 2:** The differences in the CSA and FI % of the paraspinal muscles among the different age strata and gender in the LDD and asymptomatic groups

		LDD (*n* = 370)				Asymptomatic (*n* = 327)			
		18–39 (*n* = 64)	40–59 (*n* = 102)	≥ 60 (*n* = 204)	*p* value	18–39 (*n* = 130)	40–59 (*n* = 107)	≥ 60 (*n* = 90)	*p* value
Men	Multifidus CSA (cm^2^)	21.33 ± 3.55	21.13 ± 3.08	19.64 ± 3.75	0.015*	20.31 ± 2.91	20.22 ± 2.77	18.73 ± 3.34	0.019*
	Erector spinae CSA (cm^2^)	37.23 (33.66 ~ 40.59)	35.51 (29.89 ~ 39.78)	30.11 (25.7 ~ 35.02)	< 0.001*	34.06(29.76 ~ 37.28)	33.46(28.96 ~ 38.6)	28.59(25.77 ~ 35.92)	0.012*
	Psoas CSA (cm^2^)	36.63 ± 6.48	31.57 ± 5.48	25.46 ± 5.62	< 0.001*	33.39(30.34 ~ 39.4)	30.64(26.45 ~ 36.65)	26.04(23.54 ~ 30.05)	< 0.001*
	Multifidus FI%	19.11 (15.99 ~ 22.24)	21.63 (16.65 ~ 28.44)	31.74 (27.39 ~ 36.19)	< 0.001*	13.85(12.25 ~ 17.07)	17.76(13.16 ~ 21.26)	26.68(23.78 ~ 33.83)	< 0.001*
	Erector spinae FI%	13.69 (11.57 ~ 16.1)	16.69 (12.05 ~ 22.75)	23.09 (18.27 ~ 28.95)	< 0.001*	10.07(7.57 ~ 12.66)	12.58(10.81 ~ 15.88)	19.96(16.43 ~ 23.61)	< 0.001*
Women	Multifidus CSA (cm^2^)	17.53 ± 3.20	17.05 ± 2.91	15.98 ± 2.76	0.009*	16.78 ± 2.76	16.93 ± 2.92	16.77 ± 2.80	0.944
	Erector spinae CSA (cm^2^)	24.74 (23.15 ~ 29.87)	27.00 (22.36 ~ 31.55)	25.71 (22.36 ~ 29.57)	0.162	23.77 (20.9 ~ 28.04)	24.60 (22.89 ~ 27.65)	25.59 (21.13 ~ 28.85)	0.313
	Psoas CSA (cm^2^)	20.16 (17.32 ~ 22.85)	19.98 (16.85 ~ 21.69)	16.53 (14.04 ~ 18.65)	< 0.001*	19.88 (17.46 ~ 22.79)	18.35 (16.49 ~ 20.57)	16.04 (13.81 ~ 19.11)	< 0.001*
	Multifidus FI%	25.35 (23.1 ~ 29.76)	28.98 (23.26 ~ 34.58)	36.66 (32.02 ~ 41.66)	< 0.001*	18.07(16.09 ~ 20.97)	22.68(19.84 ~ 26.93)	31.59(29.01 ~ 35.55)	< 0.001*
	Erector spinae FI%	15.8 (12.44 ~ 19.27)	21.25 (16.94 ~ 26.66)	28.97 (23.22 ~ 33.06)	< 0.001*	11.8(9.62 ~ 14.02)	15.46(13.27 ~ 19.14)	23.72(19.27 ~ 28.94)	< 0.001*

*LDD* Lumbar degenerative diseases, *CSA* Cross sectional area, *FI%* Fatty infiltration rate

*Statistical significance at the level of 0.05

### The differences in the CSA and FI % of the paraspinal muscles between LDD and asymptomatic groups in different strata and sex

**Fig. 3:**
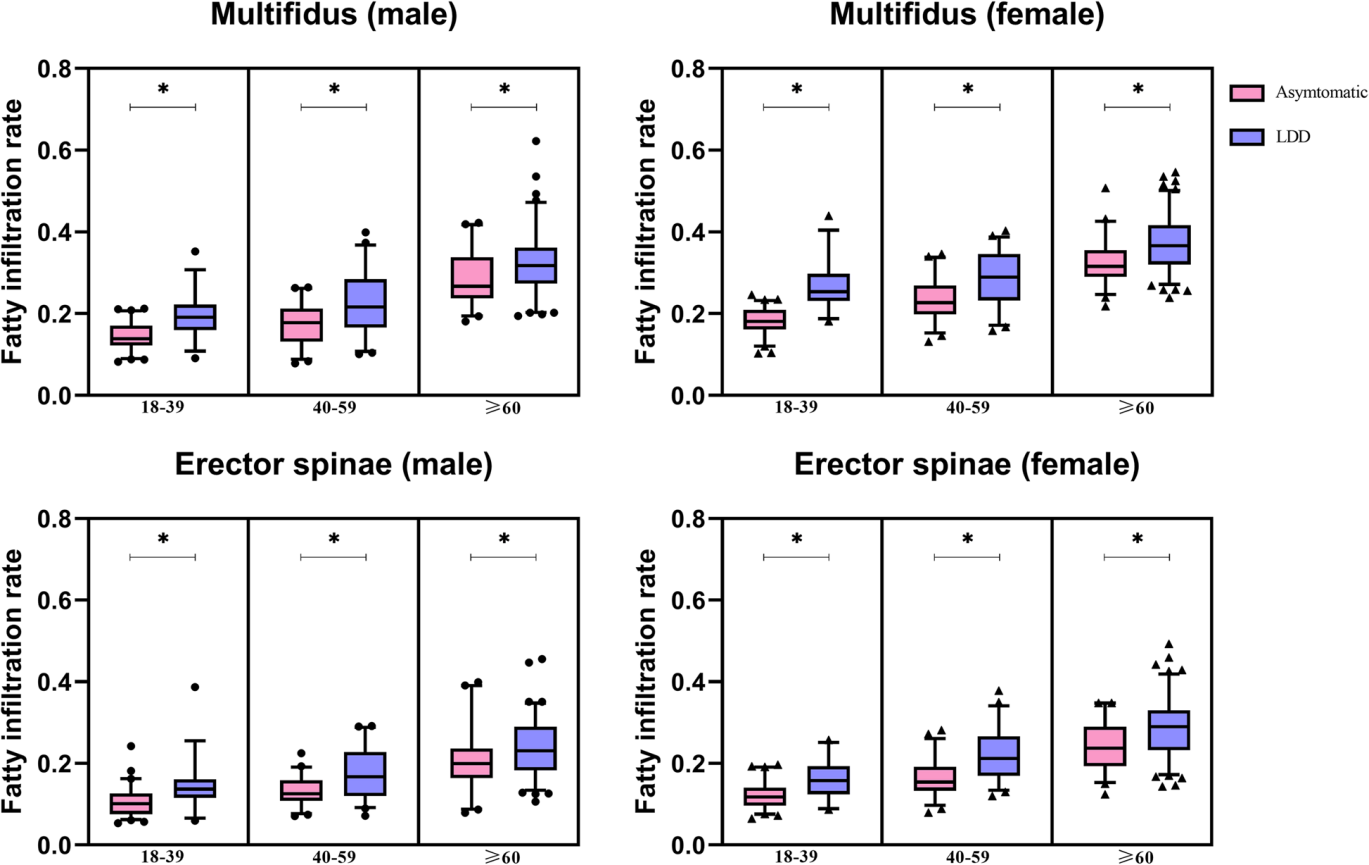
The differences in the fatty infiltration rate of the paraspinal muscles between LDD (lumbar degenerative disease) and asymptomatic groups in different strata and sex. * Statistical significance at the level of 0.05

The differences in the CSA and FI % of the paraspinal muscles between the LDD and the asymptomatic groups in different strata and sex are shown in the Table 3. There was no significant difference in the size of the paraspinal muscle cross-sectional area between the two groups. However, the FI % of the multifidus and erector spinae was significantly higher in the LDD group than that in the asymptomatic group among different age stages and sex (Fig. 3).

**Table 3:** The differences in the CSA and FI % of the paraspinal muscles between LDD and asymptomatic groups in different age strata and gender

		**18–39**			**40–59**			** ≥ 60**		
		**LDD (*n* = 64)**	**Asymptomatic (*n* = 130)**	***p* value**	**LDD (*n* = 102)**	**Asymptomatic (*n* = 107)**	***P* value**	**LDD (*n* = 204)**	**Asymptomatic (*n* = 90)**	***p* value**
Men	Multifidus CSA (cm^2^)	21.33 ± 3.55	20.31 ± 2.91	0.126	21.13 ± 3.08	20.22 ± 2.77	0.119	19.64 ± 3.75	18.73 ± 3.34	0.189
	Erector spinae CSA (cm^2^)	37.23 (33.66 ~ 40.59)	34.06 (29.76 ~ 37.28)	0.051	35.88 ± 7.96	33.77 ± 6.89	0.156	30.11 (25.70 ~ 35.02)	28.59 (25.77 ~ 35.92)	0.760
	Psoas CSA (cm^2^)	35.55 (32.49 ~ 40.10)	33.39 (30.34 ~ 3940)	0.083	31.57 ± 5.48	30.93 ± 6.04	0.577	25.46 ± 5.62	25.99 ± 5.42	0.618
	Multifidus FI%	19.21 ± 5.40	14.54 ± 3.41	< 0.001*	22.49 ± 7.83	17.42 ± 4.82	< 0.001*	31.74 (27.39 ~ 36.19)	26.68 (23.78 ~ 33.83)	0.008*
	Erector spinae FI%	13.69 (11.57 ~ 16.10)	10.10 (7.57 ~ 12.66)	< 0.001*	16.69 (12.05 ~ 22.75)	12.58 (10.81 ~ 15.88)	< 0.001*	23.09 (18.27 ~ 28.95)	19.96 (16.43 ~ 23.61)	0.009*
Women	Multifidus CSA (cm^2^)	17.53 ± 3.20	16.78 ± 2.76	0.247	17.05 ± 2.91	16.93 ± 2.92	0.836	15.98 ± 2.76	16.77 ± 2.80	0.098
	Erector spinae CSA (cm^2^)	24.74 (23.15 ~ 29.87)	23.77 (20.90 ~ 28.04)	0.147	27.00 (22.36 ~ 31.55)	24.60 (22.89 ~ 27.65)	0.097	25.97 ± 5.21	25.43 ± 5.68	0.549
	Psoas CSA (cm^2^)	20.16 (17.32 ~ 22.85)	19.88 (17.46 ~ 22.79)	0.864	19.98 (16.85 ~ 21.69)	18.35 (16.49 ~ 20.57)	0.239	16.53 (14.04 ~ 18.65)	16.04 (13.81 ~ 19.11)	0.772
	Multifidus FI%	25.35 (23.10 ~ 29.76)	18.07 (16.09 ~ 20.97)	< 0.001*	28.65 ± 6.91	23.28 ± 5.00	< 0.001*	36.66 (32.02 ~ 41.66)	31.59 (29.01 ~ 35.55)	< 0.001*
	Erector spinae FI%	15.80 (12.44 ~ 19.27)	11.80 (9.62 ~ 14.02)	< 0.001*	21.25 (16.94 ~ 26.66)	15.46 (13.27 ~ 19.14)	< 0.001*	28.66 ± 7.16	24.23 ± 5.80	< 0.001*

*LDD* Lumbar degenerative diseases, *CSA* Cross sectional area, *FI%* Fatty infiltration rate

*Statistical significance at the level of 0.05

### Results from multivariable regression models of the effect of demographic parameters and LDD on paraspinal muscles fatty infiltration.

Table 4 is the results from multivariable regression models of the effect of demographic parameters and LDD on the paraspinal muscles fatty infiltration. The results of the multiple linear regression analysis showed that the regression equation was significant. (*F* = 295.592, *p* < 0.001, adjusted R2 = 0.629). Despite BMI, age, sex, and LDD can significantly affect the paraspinal muscles fatty infiltration (*p* < 0.001). The standardized coefficients β of age, sex, and LDD were 0.627, 0.259, and 0.234 respectively.

**Table 4:** Results from multivariable regression models of the effect of demographic parameters and LDD on paraspinal muscles fatty infiltration

	B	95% CI	β	*p* value	VIF	Adjusted R^2^
Age	0.003	(0.003, 0.003)	0.627	< 0.001*	1.117	0.629
Sex	0.043	(0.035, 0.051)	0.259	< 0.001*	1.009	
BMI	0.000	(-0.001, 0.001)	0.015	0.514	1.011	
LDD	0.039	(0.031, 0.047)	0.234	< 0.001*	1.118	

*BMI* Body mass index, *LDD* Lumbar degenerative disease

*Statistical significance at the level of 0.05

## Discussion

In this study, the morphology of the paraspinal muscles were quantified by MRI in patients with LDD and asymptomatic subjects between the age of 18–85 years. We aimed to obtain the age- and sex-dependent muscle morphological differences. The population included in this study is a large sample of the Chinese populations with various age ranges. Previous studies were not based on such a large sample size of the Asian population. This study has certainly clinical significance for future studies on the Asian populations. These findings may indicate that paraspinal fat content is correlated with age, sex, and the severity of lumbar degeneration, which is an important reference for future basic research.

With aging, there exist a decreasing trend in the CSA of the paraspinal muscles in the LDD and asymptomatic groups. In a cross-sectional study involving 468 participants (aged 18–88 years) using MRI, the whole-body skeletal muscle mass was found to be negatively associated with aging [19]. Murata et al. [12] conducted a longitudinal study of 1849 individuals, and determined that the CSA of the psoas and paraspinal muscle significantly decreased over a 10-year period, which was similar to the results of the present study. Furthermore, we found that the psoas showed the most significant decreasing trend among all paraspinal muscles. On the contrary, the FI% of the paraspinal muscles gradually increased with aging. Peng et al. used the qCT imaging to investigate 516 Chinese females, and they found that the fatty infiltration of the paraspinal muscles increased with aging [20]. The erector spinae are primarily more vertical in comparison to the multifidus, which means that they might bear more pressure. This might explain why the multifidus showed more fatty accumulation than the erector spinae.

It is known that the condition of the paraspinal muscles is associated with several variables such as age, sex, and the physical condition. However, most previous studies included smaller sample sizes and did not group cohorts by age and sex to investigate the correlation between paraspinal muscles and LDD. Our study examined age- and sex-matched groups separately, and obtained results like the previous research by Crawford et al. [21]. Compared to asymptomatic males, they found that asymptomatic females displayed greater fatty infiltration and smaller CSA of the multifidus [21]. Sasaki et al. also found that males had a greater CSA and lower FI% than females in the paraspinal muscles [22]. We found that these sex-dependent differences still persisted in the LDD patients. These findings stress the importance that the effects of age and sex on paraspinal muscle morphology are critical, which should therefore be evaluated separately. Kim et al. [23] investigated 100,000 individuals and demonstrated the incidence of lumbar disc herniation was greater in women than in men, which might be explained by the sex-dependent difference in the morphology of the paraspinal muscles.

The role of the paraspinal muscles in the development of LDD has been recognized by number of studies. They suggested that the paraspinal muscles were essential to lumbar stabilization and movement [7, 8]. Researchers have found a link between multifidus and various degenerative diseases of the lumbar spine [5, 24–26]. Fortin et al. showed the fatty infiltration of the multifidus of the research group was significantly higher than that of the control group [27]. What’s more, a study found a significant correlation between the quality of the multifidus and spondylolisthesis [28]. But most of them focused on multifidus, a large-scale study on the relationship between the erector spinae and LDD is scarce. In the present study, we found that there was no significant difference in the CSA of the paraspinal muscles between the LDD and the asymptomatic groups, However, no matter the multifidus or the erector spinae, fatty infiltration in the LDD group was greater than that in the asymptomatic group. These relationships are consistent across the different age- and sex-matched groups. Teichtahl et al. reported that the FI% instead of the CSA was associated with LBP [26]. However, Agten et al. found that there was no difference in the CSA of the multifidus and the erector spinae between patients with LBP and healthy controls [29]. Paalanne et al. [30] found The CSA of the erector spinae and multifidus did not differ between the various degrees of pain clusters, D’Hooge et al. [31] found no difference in multifidus CSA between individuals with LBP and controls. Cuellar et al. [32] reported no association between muscle size and LBP in older adults in a comprehensive review. These results suggests that pathologic changes in the paraspinal muscles (decreased lean mass and increased fat mass) are the primary cause of lumbar spine degeneration while not the CSA. Though previous study demonstrated that patients with LBP show a reduction in CSA of the paraspinal muscle [4], however, previous meta-analysis of imaging studies using patients with LBP also revealed inconsistent results among studies [33, 34]. The different study population, measure methods, the number of included segments or the grouping method may lead to a systematic difference in results of CSA.

Multiple causes have been suggested for muscle degeneration [35], among which denervation might be one. Denervation is caused by compression or injury of the axons of motor neurons due to nerve compression from the foramen or spinal canal, which lead to atrophy of both fiber types with structural changes (muscle replacement by fat and connective tissue) in the multifidus [35–38]. Yoshihara et al. found significant decreases in the size of Type I (slow-twitch fibers) and Type II (fast-twitch fibers) muscle fibers at the involved level [38]. But the CSA of the muscle may not decrease due to the fatty infiltration in the muscle bundle. Another possible reason might be the disuse/muscle unloading mechanisms [39, 40]. Hides et al. found bed rest leads to preferential atrophy (decrease in the CSA) of the multifidus [39]. Bailey et al. found that multifidus structural changes differ between phases after IVD injury [40]. The inflammatory mechanisms with increased M1 macrophage proportion and elevated TNF expression play a critical role in regulating multifidus change which characterized by fibrosis, fatty infiltration, muscle fiber-type transformation and local inflammation [41]. Using Finite-Element model, Kang et al. found that the lumbar paraspinal muscles can reduce the pressure on the intervertebral disc in lumbar spine [9]. According to these results, it can be stated that there is an inseparable relationship between lumbar paraspinal muscles and LDD.

### Limitations

This study has several limitations. Firstly, we measured the paraspinal muscles at only one level, Nevertheless, the reliability of the method has been confirmed by previous studies [5, 24, 42]. Secondly, the influence of exercise was not considered. In general, individuals who perform regular exercise would be expected to have greater muscle mass. Finally, all our study participants were Chinese and could only represent characteristics of a single population.

## Conclusion

Age- and sex-dependent differences were found in the morphology and composition of paraspinal muscles between subjects with and without LDD. Further long-term follow-up investigations and basic studies will continue to confirm the natural history with aging of the paraspinal muscles and their association with LDD.

## Data Availability

The datasets generated and/or analyzed during the current study are not publicly available due to data privacy rules but are available from the corresponding author on reasonable request.
